# Ozonated Oil in Liposome Eyedrops Reduces the Formation of Biofilm, Selection of Antibiotic-Resistant Bacteria, and Adhesion of Bacteria to Human Corneal Cells

**DOI:** 10.3390/ijms241814078

**Published:** 2023-09-14

**Authors:** Valentina Gentili, Giovanni Strazzabosco, Niccolò Salgari, Alessandra Mancini, Sabrina Rizzo, Silvia Beltrami, Giovanna Schiuma, Fabio Casciano, Andrea Alogna, Daniela Passarella, Sergio Davinelli, Giovanni Scapagnini, Alessandro Medoro, Roberta Rizzo

**Affiliations:** 1 Department of Chemical, Pharmaceutical and Agricultural Science, University of Ferrara, 44121 Ferrara, Italy; valentina.gentili@unife.it (V.G.); giovanni.strazzabosco@unife.it (G.S.); sabrina.rizzo@unife.it (S.R.); silvia.beltrami@unife.it (S.B.); giovanna.schiuma@unife.it (G.S.); andrea.alogna@unife.it (A.A.); roberta.rizzo@unife.it (R.R.); 2 Department of Translational Medicine, University of Ferrara, 44121 Ferrara, Italy; slgncl@unife.it (N.S.); fabio.casciano@unife.it (F.C.); 3 Department of Ophthalmology, University of “Magna Graecia”, 88100 Catanzaro, Italy; alessandra.mancini@unicz.it; 4 Laboratory for Advanced Therapeutic Technologies (LTTA), University of Ferrara, 44121 Ferrara, Italy; 5 Department of Medicine and Health Sciences “V. Tiberio”, University of Molise, 86100 Campobasso, Italy; daniela.passarella@unimol.it (D.P.); sergio.davinelli@unimol.it (S.D.); giovanni.scapagnini@unimol.it (G.S.)

**Keywords:** antiseptic, ophthalmology, antimicrobial agents, multidrug-resistant organisms, povidone-iodine, chlorhexidine, liposomal ozonated oil, toxicity, biofilm

## Abstract

The recent attention to the risk of potential permanent eye damage triggered by ocular infections has been leading to a deeper investigation of the current antimicrobials. An antimicrobial agent used in ophthalmology should possess the following characteristics: a broad antimicrobial spectrum, prompt action even in the presence of organic matter, and nontoxicity. The objective of this study is to compare the antimicrobial efficacy of widely used ophthalmic antiseptics containing povidone-iodine, chlorhexidine, and liposomes containing ozonated sunflower oil. We determined the minimum inhibitory concentration (MIC) on various microbial strains: *Staphylococcus aureus* (ATCC 6538), methicillin-resistant *Staphylococcus aureus* (ATCC 33591), *Staphylococcus epidermidis* (ATCC 12228), *Pseudomonas aeruginosa* (ATCC 9027), and *Escherichia coli* (ATCC 873). Furthermore, we assessed its efficacy in controlling antibiotic resistance, biofilm formation, and bacterial adhesion. All three antiseptic ophthalmic preparations showed significant anti-microbicidal and anti-biofilm activity, with the liposomes containing ozonated sunflower oil with the highest ability to control antibiotic resistance and bacteria adhesion to human corneal cells.

## 1. Introduction

Intraocular procedures carry the risk of severe ocular complications including infectious endophthalmitis, which can have devastating consequences. This condition can primarily arise from bacterial infections. However, few observations have been made regarding ocular pathogens’ epidemiology and susceptibility patterns, even though antimicrobial resistance (AMR) has been recognized as a significant healthcare threat worldwide [1]. Multidrug-resistant organisms (MDRO), or microorganisms that are resistant to several different drug classes, are on the rise [2]. MDRO, like *Staphylococcus aureus* and *Pseudomonas aeruginosa*, are growing in importance in the field of ophthalmology [3]. Also, methicillin-resistant *S. aureus* (MRSA) ocular infections are increasing, according to several surveillance studies [4]. To provide coverage for the more prevalent Gram-positive (*S. aureus, Streptococcus* spp.) and Gram-negative (*P. aeruginosa, E. coli, Klebsiella*) species, empiric antibiotics are frequently recommended for these conditions prior to culture findings [5,6]. Also frequently prescribed are broad-spectrum antibiotics such as fluoroquinolones, gentamicin, tobramycin, polymixin B, and trimethoprim [7]. It is alarming to see that these antibiotics are becoming less effective against *S. aureus* and *P. aeruginosa* [8], suggesting alternative strategies.

For these reasons, the importance of using antiseptics in the perioperative phases has become increasingly crucial. Due to their proven efficacy, povidone-iodine (PVP-I) and chlorhexidine are widely used antiseptic agents in ophthalmic surgery [9,10]. Specifically, PVP-I is an iodophor, which is a water-soluble complex of iodine and a solubilizing polymer carrier called polyvinylpyrrolidone. Iodine quickly enters microorganisms and oxidizes essential macromolecules (proteins, nucleotides, and fatty acids), ultimately causing microbial death. Additionally, PVP-I exhibits a wide range of antibacterial action against fungi, protozoa, and Gram-positive and Gram-negative bacteria, including strains that are resistant to antibiotics and antiseptics [11,12]. It has also shown antimicrobial effects on enveloped and nonenveloped viruses, as well as certain bacterial spores when exposed for an extended period of time [11,13,14]. Furthermore, PVP-I has demonstrated effectiveness against in vitro and ex vivo mature bacterial and fungal biofilms [15,16]. On the other hand, chlorhexidine, a synthetic biguanide with cationic surfactant properties, exhibits a wide-ranging antibacterial effect as well as partial antifungal properties. By interfering with microbial cell membranes and inducing coagulation of cytoplasmic proteins, chlorhexidine maintains residual activity for several hours [17].

Although there is strong evidence for their efficacy and acceptable tolerability and safety in clinical practice, some adverse reactions associated with their use have been reported even at low concentrations, such as corneal epithelial toxicity, postoperative eye pain, persistent corneal epithelial defects, and an increased risk of keratitis [18,19,20]. Naor et al. demonstrated significant endothelial damage in bovine eyes when PVP-I concentrations exceeded 0.05% [21]. Moreover, although iodine allergies are rare, direct toxicity can occur, especially with repeated exposures, resulting in a high incidence of endophthalmitis [9,10]. Conversely, several other antiseptics (e.g., alcohol-containing disinfectants) are unsuitable for ophthalmic use due to their toxic effects on the corneal epithelium [16].

Given the increasing number of intravitreal injections performed each year and the importance of ocular antisepsis in intraocular procedures, alternative approaches and the development of new formulations are necessary. An ideal disinfectant should have a wide antimicrobial spectrum, rapid action even in the presence of organic matter, and be non-toxic [22]. In this context, liposomal ozonated oil represents a possible novel ocular formulation for preventing and treating ocular infections. Ozonated oils are obtained by a chemical process of ozonization of unsaturated fatty acids in vegetable oils, producing ozonated derivatives, the ozonides, which are more stable with fatty acids than ozone itself, with a relatively long lifetime [23,24]. These derivatives are highly reactive oxidants with documented bactericidal, antiviral, and antifungal activities, together with anti-inflammatory and tissue-repair properties, which can be applied to several pathologies such as skin diseases, pathologies of the vaginal mucosa, oral ulcers, periodontics, and eye infections [23,25,26,27,28,29]. Mechanistically, liposomes adhere to the surface of the pathogen, inducing its breaking through ozonolysis and releasing ozonides. These ozonides infiltrate the pathogen and undergo hydrolysis, generating oxygenated compounds like lipid peroxides and reactive oxygen species (ROS). These oxygenated compounds target proteins and lipids, as well as other macromolecules such as enzymes and DNA/RNA, inducing modifications in the pathogen’s structure and destroying it. Consequently, liposomal ozonated oil exhibits dual antibacterial actions: direct oxidation of the pathogen’s surface and alteration of structure and functions of pathogen macromolecules [23,25].

To further preserve the properties of ozonides and improve their tolerance by the ocular surface, a formulation based on liposomal sunflower ozonated oil and other ingredients has recently been designed for ophthalmic applications [30]. Indeed, sunflower oil embedded in liposomes may favor the stabilization of the lipid phase of the tear film reducing the evaporation of the aqueous phase and guaranteeing immediate relief [25]. Moreover, the ozonated oil liposomes are often included in a solution of hypromellose methylcellulose (HPMC), which is extremely biocompatible with the delicate ocular surface tissue [31,32].

To this end, this study evaluated the antimicrobial activity and the ability to reduce the selection of antibiotic-resistant bacteria and biofilm formation of a liposomal ozonated oil preparation (Ozodrop, FB Vision, San Benedetto del Tronto, Italy) confronting it with widely used ophthalmic antiseptic preparations containing PVP-I (Iodim, Medivis, Tremestieri Etneo, Italy) and chlorhexidine (Dropsept, Sooft, Montegiorgio, Italy) as active principles.

## 2. Results

### 2.1. Antimicrobial Activity

The antimicrobial activity of Ozodrop (FB Vision, San Benedetto del Tronto, Italy), an eye drop preparation containing ozonated sunflower oil liposomes, was evaluated. Ozodrop also contains HPMC, boric acid, sodium tetraborate (which has mild antiseptic and astringent characteristics), disodium edetate sodium (used to remove calcium deposits from eyes), polihexanide (PHMB), and deionized water. The efficacy of this preparation was compared with two gold standard actives: PVP-I with hyaluronic acid, and chlorhexidine with tocopherol polyethylene glycol 1000 succinate, both in eye drop preparations.

The minimal inhibitory concentration (MIC) was determined using the EUCAST microdilution method [33], with a two-fold dilution ranging from 50 to 0.3%. After 24 h of treatment, absorbance was measured and compared to untreated bacteria. Table 1 shows the MIC values obtained for each ophthalmic antiseptic preparation on corresponding bacterial strains. The three preparations were effective on Gram-positive bacteria, in a range from 12.5 to 50%. Only the ozonated sunflower oil liposome preparation showed a quantifiable MIC against Gram-negative bacteria. PVP-I and chlorhexidine preparations had the highest MIC concentration used (100%) against Gram-negative bacteria.

### 2.2. Antibiofilm Activity

Since both *P. aeruginosa* (ATCC 9027) and *S. aureus* (ATCC 6538) can produce biofilm, the ability of the three ophthalmic preparations to prevent the formation and destruction of the preexisting biofilm was evaluated. Biofilm production is a complex, multi-step process that is often associated with various bacterial species [34]. Adhesion to surfaces is a critical step in biofilm development, leading to several metabolic changes in cells such as the expression of extracellular polymeric substances (EPS) and different microbial surface components that recognize adhesive matrix molecules, including fibronectin and fibrinogen [35]. Once formed, biofilms become resistant to immune system responses and antibiotic treatment, making them nearly impossible to remove [35].

**Figure 1:**
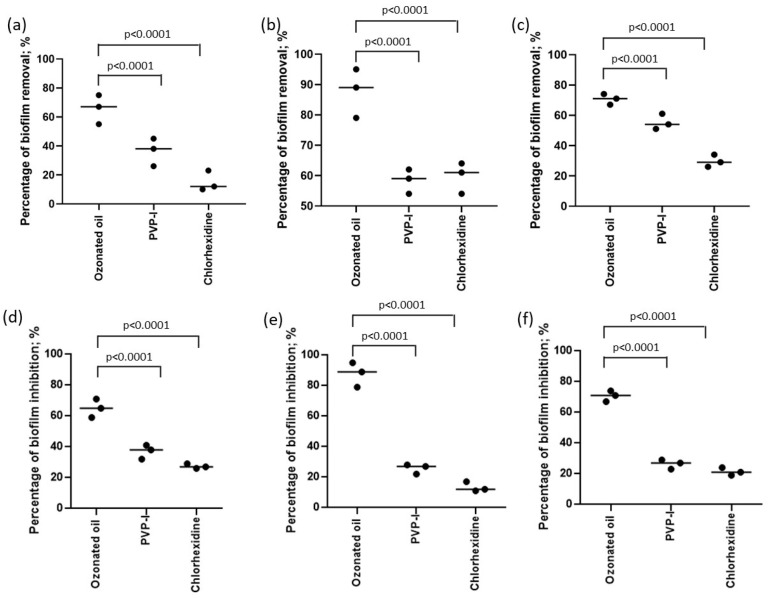
Percentage of biofilm removal in pre-formed (**a**) *P. aeruginosa* (ATCC 9027), (**b**) *S. aureus* (ATCC 6538), (**c**) MRSA (ATCC 33591) biofilm, and in forming (**d**) *P. aeruginosa* (ATCC 9027), (**e**) *S. aureus* (ATCC 6538), (**f**) MRSA (ATCC 33591) biofilm. Values are represented as the median of three experiments. *p* values were obtained by Fisher’s exact test.

This study examined the impact of three different ophthalmic solutions on eradicating pre-formed biofilm and inhibiting biofilm formation. The percentage of biomass removal was assessed using the ophthalmic solutions at their MIC values. The results showed that the ophthalmic solutions varied in their effectiveness for biofilm eradication, ranging from acceptable to excellent (Figure 1). Notably, the ophthalmic solution containing ozonated sunflower oil demonstrated superior biofilm eradication compared to the other two ophthalmic solutions in all three bacteria tested (*p* < 0.0001; Fisher’s exact test) (Figure 1a–c). Furthermore, all three ophthalmic solutions proved effective in reducing biofilm formation, with the ozonated sunflower oil solution showing the highest percentage of reduction (*p* < 0.0001; Fisher’s exact test) (Figure 1d–f).

### 2.3. Antibiotic Resistance Evaluation

*P. aeruginosa* (ATCC 9027), *S. aureus* (ATCC 6538), and MRSA (ATCC 33591) were also tested for antibiotic resistance. We selected FDA-approved antibiotics used for curing ophthalmic infectious diseases [36], including aminoglycosides (gentamicin, tobramycin, and neomycin), chloramphenicol, macrolides (azithromycin and erythromycin), quinolones (ciprofloxacin, moxifloxacin, besifloxacin, gatifloxacin, levofloxacin, and ofloxacin), and tetracyclines. The percentage of resistance levels for each bacterium to the tested antibiotic families is reported in Table 2.

**Figure 2:**
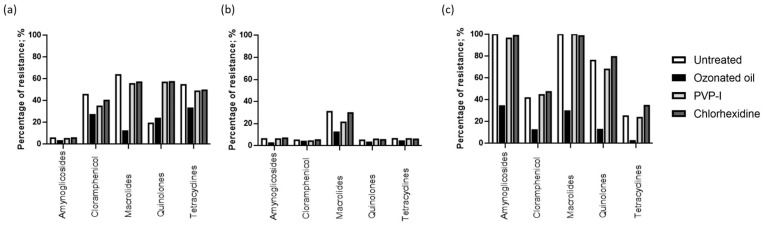
Percentage of resistant (**a**) *P. aeruginosa*, (**b**) *S. aureus*, and (**c**) MRSA to antibiotic classes, in co-treatment with the ophthalmic solutions. Values represent the mean of three experiments.

The resistance was determined by following EUCAST breakpoint values for zone diameter. We observed that *P. aeruginosa* (ATCC 9027) had a high susceptibility to aminoglycosides and quinolones (94.5% and 80.8%, respectively), and *S. aureus* (ATCC 6538) had a high susceptibility to aminoglycosides, chloramphenicol, quinolones, and tetracyclines (93.8%, 95.0%, 95.0%, and 93.6%, respectively). However, MRSA (ATCC 33591) showed low susceptibility to all the treatments. Next, we pre-treated the bacteria with the ophthalmologic solutions for 24 h before the addition of antibiotics. Treatment with the ophthalmic solution containing ozonated sunflower oil increased the susceptibility of *P. aeruginosa* (ATCC 9027) to chloramphenicol, macrolides, and tetracyclines (73.0%, 88.0%, and 67%, respectively) (*p* = 0.012; *p* = 1.6 × 10^−14^; and *p* = 0.02, respectively; Fisher’s exact test) (Figure 2a). It also increased the susceptibility of *S. aureus* (ATCC 6538) to macrolides (94.2%) (*p* = 0.00001; Fisher’s exact test) (Figure 2b). Furthermore, it increased the MRSA (ATCC 33591) susceptibility to aminoglycosides, chloramphenicol, macrolides, quinolones, and tetracyclines (65.7%, 87.7%, 70.3%, 87.4%, and 97.7%, respectively) (*p* = 8.2 × 10^−11^; *p* = 1.0 × 10^−23^; *p* = 0.000007; *p* = 4.3 × 10^−20^; and *p* = 1.6 × 10^−27^, respectively; Fisher’s exact test) (Figure 2c). The ophthalmic solution containing PVP-I increased the susceptibility of *P. aeruginosa* (ATCC 9027) to chloramphenicol (65.2%), although not significantly, but increased the resistance to quinolones (56.8%) (*p* = 1.0 × 10^−8^; Fisher’s exact test) (Figure 2a). It also increased the susceptibility of *S. aureus* (ATCC 6538) to macrolides (78.8%), although not significant (Figure 2b). The ophthalmic solution containing chlorhexidine increased the susceptibility of *P. aeruginosa* (ATCC 9027) to chloramphenicol (59.8%) (*p* = 0.05; Fisher’s exact test) and the resistance to quinolones (57.3%) (*p* = 1.0 × 10^−8^; Fisher’s exact test) (Figure 2a). It also increased the resistance of MRSA (ATCC 33591) to tetracyclines (34.6%), although this increase was not statistically significant (Figure 2c).

### 2.4. Bacteria Adhesion Test

**Figure 3:**
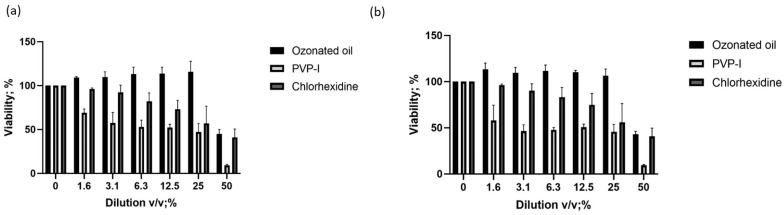
The viability of HCE-2 cells was evaluated by (**a**) MTT and (**b**) neutral red uptake after 4 h of exposure with serial dilution ranging from 50 to 1.5625% *v*/*v* of the ophthalmic antiseptic preparations. Values represent the mean ± standard deviation of three experiments.

Bacteria need to colonize their host in order to infect [37]. We evaluated the effect of bacteria with or without pre-treatment with the three ophthalmic solutions on cell adhesion in vitro. We used *P. aeruginosa* (ATCC 9027) as representative of Gram-negative, and *S. aureus* (ATCC 6538) and MRSA (ATCC 33591) as representative of Gram-positive bacteria. Firstly, we assessed the cytotoxicity of the three ophthalmic solutions at MIC concentrations. The viability of corneal epithelial HCE-2 cells was evaluated after 4 h of exposure to serial dilutions of the ophthalmic solutions. Figure 3a,b shows the results of the MTT and neutral red uptake (NRU) assays.

The viability assay revealed that the ophthalmic solution containing ozonated sunflower oil had an IC_50_ of 50% *v*/*v*, while the IC_50_ for PVP-I was 1.56%, and for chlorhexidine was 6.25% *v*/*v*. We used IC_50_ concentrations to evaluate the ability to interfere with adhesion. The basal adhesion of *P. aeruginosa* (ATCC 9027) was 5.2 × 10^5^ CFU, *S. aureus* (ATCC 6538) was 5.4 × 10^5^ CFU, and MRSA (ATCC 33591) was 5.7 × 10^5^ CFU. Co-treatment with the ophthalmic solutions reduced the bacteria adhesion. Both PVP-I and chlorhexidine preparations reduced the CFU of adherent bacteria by 1Log_10_ (*p* < 0.001; Mann–Whitney U test). However, the ophthalmic solution containing ozonated sunflower oil was able to reduce adhesion by up to 2Log_10_ (*p* < 0.0001 Mann–Whitney U test) (Table 3).

### 2.5. Ophthalmologic Solution Resistance Evaluation

*P. aeruginosa* (ATCC 9027), *S. aureus* (ATCC 6538), and MRSA (ATCC 33591) were also tested for ophthalmic solution resistance. *P. aeruginosa* (ATCC 9027), *S. aureus* (ATCC 6538), and MRSA (ATCC 33591). These three bacterial strains were exposed to the MIC of the three ophthalmologic solutions for 7 days. After 24 h of microbial incubation, the MIC values were recorded. Table 4 shows that we obtained the same MIC values for all three ophthalmic solutions of those reported in Table 1.

## 3. Discussion

In this study, we evaluated the efficacy of liposomal ozonated oil for the first time in preventing biofilm formation, eliminating pre-existed biofilm, as well as maintaining antibiotic susceptibility of different bacterial strains. We compared it with widely used antiseptic agents such as PVP-I and chlorhexidine.

Our experiments demonstrated and confirmed that all three ophthalmic antiseptic preparations exhibited microbicidal activity at different concentrations. They were effective on Gram-positive bacteria, ranging from 12.5 to 50%. Only ozonated sunflower oil embedded in liposome preparation showed a MIC against Gram-negative bacteria. The MIC for Gram-negative bacteria with PVP-I and chlorhexidine preparations was above the highest concentration used (50%). The differences in these results compared to previously published analyses may be due to different microbial strains and experimental conditions [38]. The novelty of this research lies in evaluating the effect of the three ophthalmic solutions on biofilm formation, antibiotic susceptibility of bacteria, and cell adhesion. The ability to remove pre-formed biofilm ranged from fair to excellent, with the ophthalmic solution containing ozonated sunflower oil with the highest effect compared to the other two ophthalmic solutions. This may be attributed to the liposomal component, which can counteract biofilm surfaces that can absorb antimicrobial substances, reducing their bioavailability [39].

Treatment with the ophthalmic solutions increased susceptibility to the evaluated antibiotics, with the ophthalmic solution containing ozonated sunflower oil showing a wider range of action, particularly against macrolides and chloramphenicol, for the three bacteria tested. The efficacy of the ophthalmic solution containing ozonated sunflower oil might be ascribed to the presence of liposomes, which may facilitate the delivery not only of the ozone inside the bacteria but also of the antibiotics, creating a gap in the membrane where they interact. Similarly, liposomes can interfere with bacteria adhesion to cells, as demonstrated by the 2Log_10_ reduction in adherent bacteria, when the infection is treated with the ophthalmic solution containing ozonated sunflower oil. Interestingly, we observed that a 7-day treatment with the three ophthalmic solutions did not affect the MIC concentration for all the tested bacteria, suggesting their stable efficacy after prolonged use in common clinical practice. It is important to note that this study has limitations, including the absence of clinical isolates and in vivo models, but it represents an important starting point to further investigate the potential of liposomal ozonated oils in ophthalmic applications.

Overall, these results suggest that the three ophthalmic solutions can control bacteria replication and biofilm formation. The ophthalmic solution containing ozonated sunflower oil showed a higher ability to control antibiotic resistance, bacteria adhesion, and biofilm formation, supporting the validity of its use as an antiseptic. Additionally, when applied to the ocular surface, this solution has documented regenerative and anti-inflammatory properties [23,31,40,41,42]. This implies a possible use as an adjuvant in various ophthalmic diseases characterized by inflammation, infection, and tissue damage.

## 4. Materials and Methods

### 4.1. Antimicrobial Activity

The antimicrobial activity was assessed against *S. aureus* (ATCC 6538), MRSA (ATCC 33591), *S. epidermidis* (ATCC 12228), *P. aeruginosa* (ATCC 9027), and *E. coli* (ATCC 8739). Bacterial strains were cultured at 37 °C in Mueller–Hinton broth (Liofilchem, Roseto degli Abruzzi, Italy).

For each strain, the following ophthalmic antiseptic preparations were used: Ozodrop (self-preserved ozonated sunflower oil, hydroxypropyl methylcellulose, liposomes, boric acid, sodium tetraborate, disodium edetate sodium, PHMB, deionized water—concentration not specified; FB Vision, San Benedetto del Tronto, Italy), Iodim (PVP-I 0.6%, hyaluronic acid vehicle; Medivis, Tremestieri Etneo, Italy), Dropsept (chlorhexidine 0.02%, 0.5% tocopherol polyethylene glycol 1000 succinate (TPGS1000); Sooft, Montegiorgio, Italy). Ozonated sunflower oil has been produced in accordance with patent IT201600078872A1, ensuring stability that meets the registration requirements outlined in the technical file submitted to the notification body. The product has been assessed and found to be compliant with all necessary requirements for market placement. The MIC was determined by performing the EUCAST broth microdilution method in 96-well U-bottom microplates defining it as the lowest concentration of drug that inhibits the visible growth of the organism after overnight incubation [33]. All ophthalmic antiseptic preparations were serially diluted in 1× PBS by using a 2-fold dilution ranging from 100 to 0.3% and incubated with 5 × 10^6^ CFU/mL of each microorganism.

### 4.2. Antibiofilm Effect of Using Crystal Violet (CV) Assay

#### 4.2.1. Effect on Biofilm Formation Ability

The ability of ozonated oils to prevent cell adhesion at the MIC value was tested. Briefly, 200 µL of bacterial suspension (OD 600 nm of 0.04 ± 0.02) was added to 96-well polystyrene microtiter plates and incubated at 37 °C for two hours without shaking to allow bacteria to adhere to the plate’s surface [43]. After incubation, the contents of the plates were removed, and the plates were rinsed three times in 0.9% (*w*/*v*) saline solution to remove non-adherent cells. Then, 180 µL of fresh medium and 20 µL of the ophthalmic solution at MIC value were added to adherent cells. After 24 h of incubation at 37 °C, the contents of the plates were removed, and the wells were cleaned with saline solution, and dried overnight by air.

#### 4.2.2. Effect on Established Biofilms

In a 96-well plate, each well received 200 µL of bacterial solution (OD 600 nm of 0.04 ± 0.02) and was incubated for 24 h at 37 °C with 150 rpm shaking. After the incubation period, the contents of each well were removed and cleaned with 0.9% (*w*/*v*) saline solution. Then, 20 µL of ophthalmic solution and 180 µL of new Mueller–Hinton medium were added to the 24-h-old biofilms. Control wells contained untreated Mueller–Hinton medium. The plates were once more incubated for 24 h at 37 °C and 150 rpm. After incubation, the contents of each well were removed, thoroughly cleaned with saline solution three times, and left to air dry overnight.

#### 4.2.3. Assessment of Biofilm Biomass

The adhering bacteria were fixed with 200 µL of 96% (*v*/*v*) ethanol for 15 min to measure the biofilm mass. After that, 200 µL of 0.1% crystal violet (Sigma-Aldrich, St. Louis, MO, USA) was added to each plate well, and the plate was left to stain for 10 min at room temperature. The plates were then air-dried, the excess crystal violet rinsed off and gently washed in saline solution. The crystal violet was then dissolved in 200 µL of 33% (*v*/*v*) glacial acetic acid, and the biomass was determined by measuring the OD at 570 nm using a microplate reader BioTek ELx808U (BioTek, Winooski, VT, USA).

The percentage of biomass reduction (%BR) in comparison to biofilms not exposed to ophthalmic solution was calculated as follows:

$$\%BR=\frac{{OD}_{CTR}-{OD}_{OS}}{{OD}_{CTR}}\times 100$$

where OD_CTR_ is the OD_570nm_ value of control wells and OD_OS_ is the OD_570nm_ value for the ophthalmic solution-treated wells.

### 4.3. Antibiotic Susceptibility Test

Using the traditional disk diffusion method, the Kirby–Bauer test, and commercially available antibacterial disks, selected strains were evaluated for their susceptibility or resistance to various antibiotics. Bacteria were cultured on Tryptic soy agar (Liofilchem, Roseto degli Abruzzi, Italy) for 24 h at 37 °C for the disk diffusion experiment. Harvested soy agar was then suspended in sterile water to a turbidity of 0.5 McFarland, equivalent to 1.5 × 10^8^ CFU/mL. A cotton swab was used to inoculate the samples in triplicate onto plates of Mueller–Hinton agar. After 24 h at 37 °C, the diameter of inhibitory zones was precisely determined by a precision caliper (Mitutoyo, Andover, UK). The percentage of resistance was calculated as 100 − [(Control diameter of the inhibitory zone − (Sample diameter of inhibitory zone/Control diameter of the inhibitory zone))] × 100.

### 4.4. Cytotoxicity Assay

The human corneal epithelial cell line (HCE-2) (ATCC, Manassas, VA, USA; number CRL-11135) was cultured in keratinocyte serum-free medium (Gibco, Waltham, MA, USA); Number: 17005-042) supplemented with 0.05 mg/mL bovine pituitary extract (Gibco, USA), 5 ng/mL epidermal growth factor, 500 ng/mL hydrocortisone, and 0.005 mg/mL insulin (Gibco, USA) at 37 °C and 5% CO_2_. Cell viability of HCE-2 was evaluated by performing the colorimetric MTT (3-[4,5-dimethylthiazole-2-yl]-2,5-diphenyltetrazolium bromide, Roche, Basilea, Switzerland) assay according to the manufacturer protocol. Briefly, HCE-2 cells were seeded in 96-well plates at a density of 5 × 10^3^ cells per well and cultured overnight. The day after, HCE-2 cells were treated with the ophthalmic solutions at different 2-fold serial dilutions (ranging from 50 to 1.5625% *v*/*v*) for 4 h. After incubation time, MTT was performed and the absorbance was measured at 450 nm by using a microplate reader (Multiskan FC, Thermo Fisher, Waltham, MA, USA). Cytotoxicity was calculated as IC_50_, the concentration of a given agent resulting in lethal to 50% of the cells.

### 4.5. Neutral Red Uptake (NRU) Assay

The HCE-2 cells were seeded in 96-well tissue culture plates and treated for the appropriate period. The plates were then incubated for 2 h with a medium containing neutral red (Abcam, Cambridge, UK). The cells were subsequently washed, the dye extracted in each well, and the absorbance read using a spectrophotometer (BioRad, Milan, Italy). Cytotoxicity was calculated as IC_50_, the concentration of a given agent that is lethal to 50% of the cells.

### 4.6. Bacteria Adhesion Test

The HCE-2 cells were resuspended in fresh medium supplemented with 10% serum without antibiotics at a concentration of 2 × 10^5^ cells/mL [44], as evaluated by a hemocytometer. One milliliter of cell suspension was seeded in three sets of duplicate wells (one for each strain) in the center of a 24-well plate and incubated overnight in a cell culture incubator. One isolated colony of each bacterial strain was inoculated in 5 mL of LB broth (1% tryptone, 0.5% sodium chloride, 0.5% yeast extract) and grown overnight at 37 °C with vigorous shaking (180 rpm).

The HCE-2 cells were washed with warm 1× DPBS and then added with 1 mL of fresh medium supplemented with 10% serum without antibiotics. Fresh medium without cells was used to determine the total number of bacteria in the inoculum for each strain. An aliquot (10^6^ CFU) of each bacterial culture was added to one set of duplicate wells containing HCE-2 cells (multiplicity of infection of 5:1 bacteria:cells) and to one well not containing cells. The cells were incubated for 3 h at 37 °C with 5% CO_2_. The medium was removed from the infected cells, which were washed 3 times with warm 1× DPBS. The adhered bacteria were detached with 100 μL of 1% Triton X-100 for 10 min at room temperature and then added with 900 μL of LB medium. After gentle homogenization, serial 10-fold dilutions of the suspensions of adhered bacteria were inoculated in LB broth and 100 μL from 3 dilutions was plated on LB agar and incubated overnight at 37 °C. The colonies were counted as CFU of adhered bacteria. Only plates with 10–300 colonies were counted. To ensure that 1% Triton X-100 treatment did not affect bacteria viability, we performed a control experiment, maintaining the bacteria with and without 1% Triton X-100 for 10 min and evaluated CFU, as previously reported. We did not find evidence of any difference in CFU count (Table 5).

### 4.7. Statistical Analysis

Statistical analyses were performed using GraphPad Prism software (v.8, San Diego, CA, USA). Data were statistically compared using the Mann–Whitney U test, as all the data did not display a normal distribution based on the Kolmogorov–Smirnov and D’Agostino–Pearson normality tests. Percentages were compared by Fisher’s exact test. *p* values < 0.05 were considered significant.

## Figures and Tables

**Table 1:** MIC values (% *v*/*v*) of the ophthalmic antiseptic preparations on the different microbial strains after 24 h of incubation.

Bacterial Strain	Ozonated Oil Ophthalmic Solution	PVP-I Ophthalmic Solution	Chlorhexidine Ophthalmic Solution
*E. coli (ATCC 8739)*	25%	100%	100%
*P. aeruginosa (ATCC 9027)*	50%	100%	100%
*S. aureus (ATCC 6538)*	12.5%	50%	12.5%
*MRSA (ATCC 33591)*	25%	50%	25%
*S. epidermidis (ATCC 12228)*	12.5%	50%	12.5%

**Table 2:** Percentage (%) resistance levels of the detected genera to the tested antibiotic families.

Antibiotics	*P. aeruginosa* (ATCC 9027)	*S. aureus* (ATCC 6538)	MRSA (ATCC 33591)
Aminoglycosides (gentamycin, tobramycin, neomycin)	5.5%	6.2%	100.0%
Chloramphenicol	45.5%	5.0%	41.7%
Macrolides (azithromycin, erythromycin)	63.6%	30.8%	100.0%
Quinolones (ciprofloxacin, moxifloxacin, besifloxacin, gatifloxacin, levofloxacin, ofloxacin)	19.2%	5.0%	76.0%
Tetracyclines	54.5%	6.4%	25.0%

**Table 3:** CFU of adhered bacteria on HCE-2 cells after 4 h of exposure to IC_50_ concentration of the tested ophthalmic solutions.

Ophthalmic Solution	*P. aeruginosa* (ATCC 9027) (CFU)	*S. aureus* (ATCC 6538) (CFU)	MRSA (ATCC 33591) (CFU)
Ozonated sunflower oil	1.7 × 10^3^	2.1 × 10^3^	1.9 × 10^3^
PVP-I	5.5 × 10^4^	4.6 × 10^4^	5.1 × 10^4^
Chlorhexidine	3.2 × 10^4^	2.2 × 10^4^	5.2 × 10^4^

**Table 4:** MIC values (% *v*/*v*) of the ophthalmic antiseptic preparations on the different microbial strains after 24 h of incubation. The bacterial strains were previously treated with ophthalmic solutions for a duration of 7 days.

Bacterial Strain	Ozonated Oil Ophthalmic Solution	PVP-I Ophthalmic Solution	Chlorhexidine Ophthalmic Solution
*E. coli (ATCC 8739)*	25%	100%	100%
*P. aeruginosa (ATCC 9027)*	50%	100%	100%
*S. aureus (ATCC 6538)*	12.5%	50%	12.5%
*MRSA (ATCC 33591)*	25%	50%	25%
*S. epidermidis (ATCC 12228)*	12.5%	50%	12.5%

**Table 5:** CFU count with and without 1% Triton X-100.

Solutions	*P. aeruginosa* (ATCC 9027)	*S. aureus* (ATCC 6538)
LB	5.7 × 10^4^	5.5 × 10^4^
LB + 1% Triton X-100	5.8 × 10^4^	5.6 × 10^4^

## Data Availability

The data presented in this study are available on request from the corresponding author.
